# Standardization of Epidemiological Surveillance of Rheumatic Heart Disease^[Author-notes ofac250-FM1]^

**DOI:** 10.1093/ofid/ofac250

**Published:** 2022-09-15

**Authors:** Amy Scheel, Kate M Miller, Andrea Beaton, Judith Katzenellenbogen, Tom Parks, Thomas Cherian, Chris A Van Beneden, Jeffrey W Cannon, Hannah C Moore, Asha C Bowen, Jonathan R Carapetis, for the Strep A Vaccine Global Consortium (SAVAC) Burden of Disease Working Group

**Affiliations:** Emory University School of Medicine, Atlanta, Georgia, USA; Wesfarmers Centre of Vaccines and Telethon Kids Institute, University of Western Australia, Nedlands, Western Australia, Australia; Perth Children’s Hospital, Nedlands, Western Australia, Australia; The Heart Institute, Cincinnati Children’s Hospital Medical Center, Cincinnati, Ohio, USA; Department of Pediatrics, University of Cincinnati College of Medicine, Cincinnati, Ohio, USA; Wesfarmers Centre of Vaccines and Telethon Kids Institute, University of Western Australia, Nedlands, Western Australia, Australia; School of Population and Global Health, University of Western Australia, Nedlands, Western Australia, Australia; Department of Infectious Disease, Imperial College London, Hammersmith Hospital, London, United Kingdom; MMGH Consulting, Geneva, Switzerland; CDC Foundation, Centers for Disease Control and Prevention, Atlanta, Georgia, USA; Wesfarmers Centre of Vaccines and Telethon Kids Institute, University of Western Australia, Nedlands, Western Australia, Australia; Department of Global Health and Population, Harvard T. H. Chan School of Public Health, Boston, Massachusetts, USA; Wesfarmers Centre of Vaccines and Telethon Kids Institute, University of Western Australia, Nedlands, Western Australia, Australia; Wesfarmers Centre of Vaccines and Telethon Kids Institute, University of Western Australia, Nedlands, Western Australia, Australia; Perth Children’s Hospital, Nedlands, Western Australia, Australia; Wesfarmers Centre of Vaccines and Telethon Kids Institute, University of Western Australia, Nedlands, Western Australia, Australia; Perth Children’s Hospital, Nedlands, Western Australia, Australia

**Keywords:** rheumatic heart disease, *Streptococcus*, surveillance

## Abstract

Rheumatic heart disease (RHD) is a long-term sequela of acute rheumatic fever (ARF), which classically begins after an untreated or undertreated infection caused by *Streptococcus pyogenes* (Strep A). RHD develops after the heart valves are permanently damaged due to ARF. RHD remains a leading cause of morbidity and mortality in young adults in resource-limited and low- and middle-income countries. This article presents case definitions for latent, suspected, and clinical RHD for persons with and without a history of ARF, and details case classifications, including differentiating between definite or borderline according to the 2012 World Heart Federation echocardiographic diagnostic criteria. This article also covers considerations specific to RHD surveillance methodology, including discussions on echocardiographic screening, where and how to conduct active or passive surveillance (eg, early childhood centers/schools, households, primary healthcare), participant eligibility, and the surveillance population. Additional considerations for RHD surveillance, including implications for secondary prophylaxis and follow-up, RHD registers, community engagement, and the negative impact of surveillance, are addressed. Finally, the core elements of case report forms for RHD, monitoring and audit requirements, quality control and assurance, and the ethics of conducting surveillance are discussed.

## Teaser

This article presents case definitions for clinical rheumatic heart disease and best-practice protocols for conducting epidemiological surveillance for rheumatic heart disease in a variety of settings.

## DISEASE CHARACTERISTICS

Rheumatic heart disease (RHD), the long-term sequela of acute rheumatic fever (ARF), classically begins with *Streptococcus pyogenes* (Strep A) pharyngitis, with emerging data also suggesting that skin infection may be a trigger [1]. ARF develops after an inappropriate immune response to streptococcal infection in a genetically susceptible host, inducing an autoimmune response that damages the valvular endothelium, predominantly on the left side of the heart. While acute rheumatic valvulitis is often reversible, a single severe ARF episode or repeated episodes of ARF often lead to permanent scarring and valvular dysfunction known as RHD. Approximately 60% of patients who experience at least 1 episode of ARF will develop RHD [2].

RHD affects >40 million people worldwide and is responsible for 300 000 deaths each year [3]. More than 80% of cases of RHD are in persons 15–49 years of age, with a higher prevalence reported among women [4]. In high-income countries, RHD has been nearly eradicated. Despite continued research into the pathogenesis of RHD and the importance of recognizing and treating Strep A infections, RHD remains a leading cause of morbidity and mortality in young adults in resource-limited or low- and middle-income countries (LMICs) [5]. Deaths attributed to RHD occur almost exclusively in persons in LMICs [6], with the highest prevalence and age-standardized mortality due to RHD found in Oceania, South Asia, and central sub-Saharan Africa [6]. Additionally, some resource-limited subpopulations in high-income countries, such as Indigenous people, continue to experience high RHD burden [7–9].

A long asymptomatic phase typically occurs between initial ARF and the development of clinical RHD, sometimes lasting decades. Up to 75% of children and young adults diagnosed with RHD do not recall an episode consistent with ARF [10–12]. RHD is often diagnosed in resource-limited settings when patients present with severe valvular involvement and cardiovascular complications, including heart failure, stroke, infective endocarditis, arrhythmias, pregnancy-related complications, and even sudden death [13]. The fact that many patients diagnosed with ARF and RHD have no recollection of a previous Strep A pharyngitis highlights the importance and potential impact of a Strep A vaccine as a primary prevention measure.

## OBJECTIVES OF SURVEILLANCE FOR RHD

An effective surveillance system for RHD serves to monitor trends in: (1) age- and sex-specific prevalence and geographical distribution of RHD; (2) the demographics and clinical characteristics of patients with RHD; and (3) disease burden estimates.

### Secondary Objectives

Surveillance systems may also aim to: (1) contribute to describing the natural history of RHD; (2) quantify different measures of disease burden, including economic (eg, costs of primary caretaker time, treatment regimens, clinic or hospital stays, and diagnostic tests), social, educational, and emotional burdens; (3) facilitate assessment of the value of interventions, including vaccination; and (4) monitor trends in prevalence to inform vaccine development and postlicensure vaccine implementation.

## CASE DEFINITIONS AND FINAL CASE CLASSIFICATIONS

Standardized case definitions are important for obtaining accurate surveillance data, enabling comparisons of surveillance data across jurisdictions, and monitoring the impact of interventions. The definitions and methods presented here may also be used as clinical endpoints for vaccine efficacy trials and for postlicensure effectiveness studies. The following case definitions for RHD have been drawn from an international advisory group, which was formed in 2009 under the auspices of the World Heart Federation (WHF) and comprised experts in RHD screening and echocardiographic manifestations of RHD [14].

For the purpose of surveillance, we propose the case definitions and classifications for RHD found in Table 1.

**Table 1.** Case Definitions of Rheumatic Heart Disease for Surveillance

Category	Case Definition
**Clinical RHD** (for use in symptomatic patients with no history of ARF)	Echocardiographic evidence (Supplementary Appendix 1) of RHD in a person who does not have concurrent signs or past history of ARF People with symptoms suggestive of ARF (see ARF surveillance protocol) should be managed accordingly and reevaluated for the presence of RHD once active rheumatic inflammation has subsided
**Clinical RHD** (for use in symptomatic patients with a history of ARF)	Echocardiographic evidence of RHD in a symptomatic person with a recent or past history of ARF after acute inflammation has subsided, as determined by normalization of inflammatory markers (ESR and CRP)
**Latent RHD** (for use in asymptomatic patients discovered during screening)	Echocardiographic evidence of RHD in an asymptomatic person discovered during echocardiographic screening
**Suspected RHD** (for use in symptomatic patients with a history of ARF and where echocardiography is unavailable)	Persistence of a pathological murmur in a patient with recent or past history of ARF after acute inflammation has subsided, as determined by normalization of inflammatory markers (ESR and CRP)

Abbreviations: ARF, acute rheumatic fever; CRP, C-reactive protein; ESR, erythrocyte sedimentation rate; RHD, rheumatic heart disease.

### Case Classification

Clinical and latent RHD cases should be classified as *definite* or *borderline* according to the 2012 WHF echocardiographic diagnostic criteria, or any future consensus modification of these criteria [14]. Cases of RHD can be further divided into subcategories using these criteria to reflect various disease patterns, as detailed in the 2012 WHF diagnostic criteria (Supplementary Appendix 1). These criteria should only be applied to people who do not have concurrent ARF [15] (see https://doi.org/10.1093/ofid/ofac252). Before diagnosing RHD, congenital, acquired, and degenerative heart disease must be considered as possible causes of mitral and aortic valve abnormalities. It is important to note that persons with congenital heart disease may also acquire RHD and should be diagnosed and treated as such. Congenital heart disease that involves the left-sided valves (such as bicuspid aortic valve and mitral valve prolapse) can be particularly challenging to distinguish from RHD and requires expert consultation if there is a concern for both concurrently (Supplementary Appendix 2).

## TYPES OF SURVEILLANCE RECOMMENDED

The selection of surveillance strategies depends on specific epidemiologic and clinical characteristics of the disease outcome of interest, the overall surveillance objectives, surveillance location, services accessibility, and the resources available to conduct surveillance (see Supplementary Appendix 3 for key surveillance definitions). For example, in resource-poor settings, the echocardiographic equipment and trained personnel required for active surveillance may not be available, and case-finding activities may be limited. Given that persons in resource-poor settings are often most at risk for RHD, surveillance is an important component of disease monitoring and control. Reliable burden estimates will inform the public health response to RHD, advocate for vaccine use, and enable monitoring of the effect of interventions. Minimal and enhanced surveillance strategies for RHD are described in Table 2 to provide guidance for those with limited resources and those with greater capacity, respectively.

**Table 2.** Surveillance Strategies for RHD

*Minimum Surveillance*
Minimal surveillance for RHD includes passive surveillance of primary healthcare facilities. Passive surveillance is based on clinical or documented ARF history, symptoms and persistent murmur, echocardiography results, or diagnosis recorded in health facility databases. Settings include primary healthcare clinics such as outpatient clinics, doctor’s offices, and hospitals. Participants are those who present to healthcare or other relevant settings on their own accord. If the provider or surveillance officer determines that the case definition for RHD has been met, it can be recorded in electronic medical records (EMRs), or a report provided to the surveillance system or local public health authorities. In the absence of availability of echocardiography, participants should be referred to a tertiary center for further testing when possible. Standard case report forms may be provided to the health facilities for completion and submission to the surveillance program. Passive surveillance for RHD is appropriate when a minimum estimate of disease burden is considered adequate for surveillance purposes, the population at risk is well-characterized demographically, and bias away from mild cases is acceptable for the purposes of the surveillance being undertaken [16].
*Enhanced Surveillance*
Enhanced surveillance of RHD includes prospective active case finding and echocardiographic confirmation among a large and well-defined population. Well-defined echocardiography protocols should be established prior to surveillance and remain constant throughout the surveillance period. Participants should be followed prospectively (monthly for antibiotic prophylaxis and annually for repeat echocardiograms) for a defined period of time using standard methods to collect demographic, clinical information, and echocardiographic images. Audits should be performed biannually to assess the completeness of case ascertainment, accuracy, timeliness, and echocardiographic images. Regular feedback of data/information is provided to healthcare workers and others involved in the surveillance process. This critical communication engages healthcare workers in the process and informs their clinical practice.

A quality management plan should be written before the start of surveillance to establish and ensure the quality of processes, data, and documentation associated with surveillance activities. Furthermore, all surveillance should be conducted in accordance with ethical guidelines (Supplementary Appendix 4).

## CASE ASCERTAINMENT AND SURVEILLANCE SETTINGS

For each data source, surveillance staff should (1) know the purpose of the data source and whether data have been routinely collected as part of patient care, mandatory collection of data under legal mandates, collected for research purposes, or other; (2) identify any legal mandates governing the operations of the data source that may affect the accessibility or quality of data from that source; and (3) describe the representative population for the data. Case ascertainment may be active or passive (Supplementary Appendix 5). Additional guidance for conducting active surveillance through echocardiographic screening can be found in Supplementary Appendix 6.

### Schools

Most screening studies for RHD have occurred in schools because schools offer a logistical advantage of surveying a large number of children in a single location. In addition, ARF incidence peaks among school-aged children, making schoolchildren ideal candidates for ARF screening. However, because RHD prevalence peaks among older teenagers and young adults, relying on schools as the sole site for surveillance will fail to incorporate the higher-risk age groups and underestimate the true disease burden. If feasible, school surveillance should be complemented by settings that capture older children and young adults. In a population with high levels of school absenteeism, surveying school attendees will lead to selection bias and usually result in an underestimate of disease burden, as factors associated with school nonattendance (often related to poverty and/or ill health) may be related to the risk of RHD. The bias should be acknowledged and school attendance rates cited; if possible, attempts should be made to survey school nonattenders, although this is more difficult and costly.

### Households

Active surveillance of households can identify persons with RHD who are unable to attend school or health services, possibly due to lack of time, financial constraints, or accessibility challenges [17]. Household surveillance has the added benefit of including young adults who are no longer at school but still at risk for RHD. Household surveillance also provides the data required to determine the population at risk and calculate the overall disease burden. Population-based household surveillance reduces the bias that arises from inequalities in access to school and healthcare; however, such surveillance is resource and time intensive. In many areas where RHD is prevalent, it is common for family members to travel for weeks at a time for employment, so these groups can be missed. Additionally, performing household screening can miss family members who attend school or work during the day. Local and cultural schedules and customs need to be considered to maximize the impact of household surveys for active surveillance.

### Primary Care

Primary care settings can be used for active and passive surveillance. In such settings, active surveillance involves systematic and unbiased screening of all consenting patients during their regular primary care visits in endemic areas. Patients identified as increased risk for RHD through initial screening, including known history of ARF, heart failure in persons aged <40 years, previous stroke, pathological murmur, palpitations, or first-degree relative with RHD should then be referred for further workup and echocardiographic testing. Active surveillance is costly, resource intensive, and relies on the engagement of primary carers and primary practitioners to maintain adequate retention rates to complete lengthy surveillance studies.

Passive surveillance in primary care settings involves recording data on patients who present to primary healthcare clinics. While often limited in diagnostics, primary care centers can play a pivotal role by contributing data on adverse outcomes and case fatalities as they manage patients in the outpatient setting after diagnosis. EMRs can assist surveillance, allowing data extraction at regular intervals. Therefore, we recommend that surveillance systems incorporate passive surveillance through medical record data (Supplementary Appendix 7).

## SURVEILLANCE POPULATION

A surveillance protocol should clearly describe enrollment eligibility criteria. Most protocols would benefit from surveying persons aged 5–30 years; however, age eligibility can vary between sites, depending on local needs and capacity. Children already receiving prophylactic antibiotics for any cause (eg, sickle cell, human immunodeficiency virus, surgical procedures) should not be excluded from RHD surveillance. However, the use of prophylactic antibiotics should be recorded. Unless specifically relevant to the surveillance aims, persons with underlying immunocompromise or chronic diseases should be included in RHD surveillance.

The surveillance population includes all eligible at-risk people from which cases of RHD are identified. This population, or denominator, must be well-characterized *a priori* to derive meaningful disease burden estimates. Without an accurate account of all people in the population who could potentially be evaluated for RHD, disease estimates may be under- or overestimated [18, 19].

Some settings allow population-wide data on disease burden to be recorded and analyzed. Examples include household surveillance in a representative sample in a community or healthcare setting that serves the entire community. In these cases, the surveillance population would be defined as all eligible people who reside in the community. Data accuracy must be assured if government-derived census data are used to determine the community’s demographic profile, such as the number of people in relevant age categories. Ongoing, multiyear surveillance might be necessary to generate reliable burden estimates if surveillance extends over a long period of time or if the population is not stable because of mobility or other logistic factors.

In instances where select primary healthcare facilities serve a portion of a population residing in the geographical catchment area, healthcare utilization surveys can be used to estimate the denominator corresponding to the cases of interest, improving the accuracy of disease burden estimates and enabling rate calculations [20]. The denominator is the number of patients within the geographical catchment area who would be expected to attend that primary healthcare facility if they developed signs and symptoms of RHD. Cases not residing in the defined catchment area should be excluded.

When undertaking surveillance in a sample of schools and/or classrooms, the surveillance population is the number of children who agree, and have parental or guardian appropriate consent, to participate in surveillance. The results can be generalized to the entire community if schools and classes are randomized at the start of surveillance or appropriate demographic characteristics of participants can be weighed against the characteristics of the catchment population.

## SPECIAL CONSIDERATIONS FOR RHD SURVEILLANCE

### Administrative Database Review

Codes used to identify RHD in EMRs are shown in Table 3. It is important to note that ARF and RHD have the same code using the *International Classification of Primary Care*, second edition (*ICPC-2*) system and would require additional information available in the EMR, including echocardiography results, to appropriately distinguish between ARF and RHD.

**Table 3.** Specific Codes for Pharyngitis in Electronic Medical Record Databases

**Type of Healthcare System**	**Rheumatic Heart Disease Code**
Primary healthcare system	
*International Classification of Primary Care*, version 2 (*ICPC-2*)	K71 (rheumatic fever/heart disease)
Hospital data system	
*International Statistical Classification of Diseases and Related Health Problems, Tenth Revision* (*ICD-10*)	I05 (rheumatic mitral valve diseases) I06 (rheumatic aortic valve diseases) I07 (rheumatic tricuspid valve diseases) I08 (multiple valve diseases) I09 (other rheumatic heart diseases)

### Registers for RHD

RHD registers have a central role in supporting prophylaxis delivery, facilitating ongoing care delivery for people living with RHD, and program evaluation. They can also be used for research, managing surgical waiting lists, and providing focused education support to people with a history of ARF or living with RHD. Given their role in patient follow-up over time, RHD registers also provide natural history data for RHD and complications secondary to RHD. We encourage the implementation of an RHD register in areas undergoing surveillance to facilitate follow-up, administer secondary prophylaxis, and contribute to natural history data.

### Pregnant Women

It is recommended that active surveillance in areas where RHD remains endemic be implemented among pregnant women. Indirect causes of maternal mortality, including heart disease, are on the rise in many LMICs and it is estimated that RHD is responsible for 11% of indirect maternal deaths [21–23]. Active surveillance among pregnant women offers a unique opportunity as this population is often connected to the healthcare system via routine antenatal care visits and provides an automatic denominator if screening is routine. All pregnant women at high risk of RHD should have routine echocardiography at least once at >20 weeks’ gestation [24].

### Implications for Secondary Prophylaxis and Follow-up

Prior to implementing an active surveillance program, the availability of secondary prophylaxis in case ascertainment settings, particularly if undertaking screening, should be determined. A complete understanding of local healthcare system infrastructure is vital to guarantee the availability of necessary administration supplies and trained healthcare workers for administering benzathine penicillin G (BPG). It is not appropriate to implement active surveillance for RHD if there is no capacity or prospects for developing capacity or delivering secondary prophylaxis to individuals diagnosed.

If active surveillance is implemented, the surveillance team must predetermine when and how monthly intramuscular BPG prophylaxis should be given. All persons diagnosed with RHD, (borderline and definite) at ongoing risk of ARF (depending on age and severity, in keeping with by local guidelines) should be offered monthly prophylaxis. Injection of BPG every 4 weeks is the recommended prophylactic regimen for secondary prevention in most circumstances. A 3-week dosing regimen is recommended for patients who have recurrent ARF [25]. In addition, all individuals diagnosed with RHD should be educated on Strep A pharyngitis and impetigo symptoms and counseled to seek medical treatment if symptoms arise to prevent recurrent episodes of ARF. For persons with a penicillin allergy, erythromycin 250 mg (in children, 10 mg/kg up to 250 mg) every 12 hours can be used [24].

### Community Engagement

Community engagement during each step of surveillance helps provide a considered approach to surveillance. Meaningful engagement can help ensure that the project is of value to the community and that the community members have an opportunity to express their values and concerns and develop a degree of ownership. The time required to forge relationships between surveillance staff and communities should not be underestimated and must be built into the surveillance protocol.

The level of community involvement in the design, implementation, monitoring, and evaluation of surveillance will depend on the resources available and community capacity. Key stakeholders can include community members, teachers, Indigenous/community healthcare workers, local healthcare services, community clinics, nurses, and general practitioners.

The potential benefits of involving the community in surveillance studies of RHD include (1) identifying the myths surrounding echocardiography/RHD that exist in the community; (2) more robust surveillance implementation following feedback from local community leaders; (3) increased community acceptance of surveillance activities; (4) increased community buy-in and utilization of available healthcare services and follow-up appointments; and (5) increased health literacy about RHD and its causes, thus encouraging community action around environmental health and social determinants.

### Negative Impact of Surveillance

It is important to acknowledge that active surveillance programs for RHD inevitably cause some harm. While a negative screening echocardiogram has not been associated with a negative impact, receiving a diagnosis of RHD has been associated with increased anxiety, decreased physical activity, and decreased child and parental perception of quality of life [26]. Peer support groups for children diagnosed with RHD can normalize quality-of-life scores and should be considered by investigators implementing screening programs [27].

It is imperative that investigators prioritize understanding the cultural and social needs in the screening location to minimize any negative impacts of the screening program. Involving community interviewers prior to initiating the surveillance program is recommended to elucidate community understanding of RHD and surveillance programs as well as beliefs surrounding RHD. In Uganda, for example, many community members cited anticipated pain and injury as a reason for not wanting to pursue an ultrasound [28]. By revealing this community belief, screening staff could alleviate these fears through educational posters and demonstrations prior to screening.

### Measurement of Disease Burden

Disease burden of RHD is typically described with prevalence. The prevalence of RHD from active surveillance screening surveys is calculated by determining the number of individuals diagnosed with RHD (numerator) divided by the total number of individuals screened (denominator). The prevalence of RHD from passive surveillance is calculated by determining the number of individuals diagnosed with RHD divided by the total number of individuals served in a health facility’s catchment area. Additional breakdowns that characterize RHD in a population should also be reported and broken down by age group and sex, including the prevalence of individuals categorized as borderline and definite RHD and those who meet the criteria for moderate or severe RHD.

Prevalence should be expressed in 5-year age groupings (ie, 5–9, 10–14, 15–19, 20–24 years of age, etc) to enable comparisons across protocols and geographic areas and the use of local census data in developing countries.

## DATA COLLECTION AND CASE REPORT FORMS

Case report forms should be used to collect only the information required to achieve the surveillance objectives. See Supplementary Appendix 8 for a list of recommended and optional variables for inclusion in all case report forms. Case report forms can be paper based, but secure electronic data forms are increasingly used. Electronic case report forms offer a number of benefits such as early detection of cases and timely information flow, a relatively inexpensive cost to operate, and improved data quality (accuracy and data completeness) via imbedded validation checks.

### Consent

Before initiating an assessment and collecting data or specimens, consent for participation in the surveillance program may need to be obtained based on the determination of an institutional review board. For children, consent needs to be obtained from their parent or legal guardian, and before examining, permission must be requested from the child (assent). Consent should be voluntary and based on sufficient information and an adequate understanding of the proposed surveillance program and the implications of participation. Flip charts and interpreters may help improve information delivery so that participants are clear about what they are consenting to. If consent is not obtained, do not proceed. For prospective active surveillance programs, each participant must be informed that participation in the project is voluntary and that they are free to withdraw, without justification, from the surveillance system at any time without consequences. The age at which consent can and should be given by the child will vary between countries/jurisdictions. It is the responsibility of surveillance staff to confirm the requirements of local, regional, or national authorities. Informed consent may be obtained for surveillance/throat examination, photos of throat, administration of throat swabs, and storage of swabs for future use such as genetic sequencing and transcriptome analysis.

General surveillance information includes unique identifier, date and time of first enrollment or echocardiogram, and site where participant is seen (eg, setting, location, postcode, state/province/region, and country). Each encounter should also record a surveillance visit number/echocardiogram number if multiple echocardiograms are performed.

Key demographic information includes date of birth or age in years (if date of birth not available), sex, ethnic origin/race, residential postcode, state, and country.

Clinical and epidemiologic information includes signs and symptoms, epidemiologic risk factors, WHF diagnostic category, details of prescribed antibiotic prophylaxis and adherence, heart failure medications, and details of cardiac catheterization and surgical procedures (if applicable).

## Supplementary Data

Supplementary materials are available at *Open Forum Infectious Diseases* online. Consisting of data provided by the authors to benefit the reader, the posted materials are not copyedited and are the sole responsibility of the authors, so questions or comments should be addressed to the corresponding author.

## Supplementary Material

ofac250_Supplementary_Data
