# Supplementary material for: Standardization of Epidemiological Surveillance of Rheumatic Heart Disease
Source: Open Forum Infect Dis. 2022 Sep 15;9(Suppl 1):S50–6. doi: 10.1093/ofid/ofac250 (PMC9474940; doi:10.1093/ofid/ofac250)
Supplement: ofac250_Supplementary_Data [file ofac250_supplementary_data.docx]

**Standardization of Epidemiological Surveillance of Rheumatic Heart Disease**

Supplementary Appendices

Table of Contents

[Appendix 1: 2012 World Heart Federation Criteria^1^ for the Diagnosis of Rheumatic Heart Disease (RHD) 2](#_Toc112332127)

[Appendix 2: Important Considerations in Applying the WHF Criteria 3](#_Toc112332128)

[Appendix 3: Comparisons of Advantages and Disadvantages of Active and Passive Surveillance 4](#_Toc112332129)

[Appendix 4: Good Practice and Ethical Considerations 5](#_Toc112332130)

[Appendix 5: Definitions of Key Surveillance Terms 7](#_Toc112332131)

[Appendix 6: Echocardiographic Screening for Rheumatic Heart Disease (RHD) 8](#_Toc112332132)

[Appendix 7: Administrative Health Databases 10](#_Toc112332133)

[Appendix 8: Variables for Inclusion in Rheumatic Heart Disease (RHD) Surveillance Datasets 11](#_Toc112332134)

## Appendix 1: 2012 World Heart Federation Criteria^1^ for the Diagnosis of Rheumatic Heart Disease (RHD)

| **Echocardiographic criteria for individuals aged ≤20 years** | |
| --- | --- |
| *Definite RHD (either A, B, C, or D)* | |
| A) Pathological MR and at least two morphologic features of RHD of the MV | |
| B) MS mean gradient ≥4 mm Hg^a-^ | |
| C) Pathological AR and at least two morphologic features of RHD of the AV^b^ | |
| D) Borderline disease of both aortic and mitral valves^c^ | |
| *Borderline RHD (either A, B, or C)* | |
| A) At least two morphological features of RHD of the MV without pathological MR or MS | |
| B) Pathological MR | |
| C) Pathological AR | |
| **Echocardiographic criteria for individuals aged >20 years** | |
| *Definite RHD (either A, B, C, or D)* | |
| A) Pathological MR and at least two morphological features of RHD of the MV | |
| B) MS mean gradient ≥4 mmHg^a^ | |
| C) Pathological AR and at least two morphological features of RHD of the AV, only in individuals aged <35 years^b^ | |
| D) Pathological AR and at least two morphological features of RHD of the MV | |
| **Echocardiographic criteria for pathological regurgitation (all four Doppler criteria must be met)** | |
| *Pathological MR* | *Pathological AR* |
| 1. Seen in two views | 1. Seen in two views |
| 2. In at least one view, jet length ≥2 cm^d^ | 2. In at least one view, jet length ≥1 cm^d^ |
| 3. Velocity ≥3 m/s for one complete envelope | 3. Peak velocity ≥3 m/s in early diastole |
| 4. Pan-systolic jet in at least one envelope | 4. Pan-diastolic jet in at least one envelope |
| **Morphological features of RHD** | |
| *Mitral valve* | *Aortic valve* |
| 1. AMVL thickening^e^ ≥3mm (age-specific)^f^ | 1. Irregular or focal thickening^i^ |
| 2. Chordal thickening | 2. Coaptation defect |
| 3. Restricted leaflet motion^g^ | 3. Restricted leaflet motion |
| 4. Excessive leaflet tip motion during systole^h^ | 4. Prolapse |

MR, mitral regurgitation; MV, mitral valve; MS, mitral stenosis; AR, aortic regurgitation; AV, aortic valve; AMVL, anterior mitral valve leaflet

^1^Remenyi B, Wilson N, Steer A, et al. World Heart Federation criteria for echocardiographic diagnosis of rheumatic heart disease--an evidence-based guideline. *Nature reviews Cardiology.* 2012;9(5):297-309.

## Appendix 2: Important Considerations in Applying the WHF Criteria (adapted from Horton et al.,)

| ^a^Congenital MV anomalies must be excluded. In adults, inflow obstruction due to nonrheumatic mitral annular calcification must be excluded. |
| --- |
| ^b^ Bicuspid AV, dilated aortic root, and hypertension must be excluded. |
| ^c^Combined AR and MR in high prevalence regions and in the absence of congenital heart disease is considered rheumatic. |
| ^d^A regurgitant jet length should be measured from the vena contracta to the last pixel of regurgitant color (blue or red). |
| ^e^AMVL thickness should be measured during diastole at full excursion. Measurement should be taken at the thickest portion of the leaflet, including focal thickening, beading, and nodularity. Measurement should be performed on a frame with maximal separation of chordae from the leaflet tissue. Valve thickness can only be assessed if the images are acquired at optimal gain settings without harmonics and with a frequency of >2.0 MHz. |
| ^f^Abnormal thickening of the AMVL is age-specific and defined as: ≥3 mm for individuals aged ≤20 years, ≥4 mm for individuals aged 21–40 years; and ≥5 mm for individuals aged >40 years. Valve thickness measurements obtained using harmonic imaging should be interpreted cautiously, and ≤4 mm thickness should be considered normal in those aged ≤20 years. |
| ^g^ Restricted leaflet motion of the anterior or posterior MV leaflet usually results from chordal shortening or fusion, commissural fusion, or leaflet thickening. |
| ^h^ Excessive leaflet tip motion results from elongation of the primary chords and is defined as displacement of the tip or edge of an involved leaflet toward the left atrium, resulting in abnormal coaptation and regurgitation. Excessive leaflet tip motion does not need to meet the standard echocardiographic definition of MV prolapse disease, as that refers to a different disease process. This feature applies to only those <35 years of age. In the presence of a flail MV leaflet in the young (<20 years of age), this single morphologic feature is sufficient to meet the morphologic criteria for RHD (that is, where the criteria state “at least two morphologic features of RHD of the MV,” a flail leaflet in a person <20 years of age is sufficient). |
| ^i^In the parasternal short-axis view, the right and noncoronary aortic cusp closure line often appears echogenic (thickened) in healthy individuals; this should be considered normal. |

MV, mitral valve; AV, aortic valve; AR; aortic regurgitation; MR; mitral regurgitation; AMVL; anterior mitral valve leaflet

From: Horton A, Gentles T, Remenyi B. Chapter 5 - Clinical Evaluation and Diagnosis of Rheumatic Heart Disease. In: Dougherty S, Carapetis J, Zühlke L, Wilson N, eds. *Acute Rheumatic Fever and Rheumatic Heart Disease.* San Diego (CA): Elsevier; 2021:69-106.

## Appendix 3: Comparisons of Advantages and Disadvantages of Active and Passive Surveillance

| **Advantages** | **Disadvantages** |
| --- | --- |
| ***Active surveillance*** |  |
| - Sensitive system that facilitates early detection of new cases, contributing to prevention of post-infection sequalae - Higher case ascertainment rate - More accurate identification of cases - Ability to verify information in the case of missing data or suspected data entry errors - Data collected can be comprehensive and specific to the surveillance objectives - Can evaluate the quality and effectiveness of case-finding process, thus minimizing selection bias - Allows real-time analysis and ability to respond/modify approach to surveillance and care - Can promote disease awareness and good health practices | - Can be costly and resource-intensive - Requires dedicated surveillance staff and/or extensive training and upskilling - Can be demanding on surveillance sites - Barriers to accessing communities (e.g., distance/cultural barriers) |
| ***Passive surveillance*** |  |
| - Can be conducted retrospectively - Requires fewer resources than active surveillance - Can support real-time reporting | - Responsibility for reporting new cases lies with the healthcare workers/laboratory staff; thus, it can be difficult to ensure consistency of reporting by healthcare providers - Difficulties caused by lack of standardization in terms of case definitions and coding - Tends to under-report disease - Often difficult to confirm data recording or entry errors retrospectively - Commonly associated with incompleteness of data recording or of microbiological studies - Vulnerable to bias due to differences in physicians’ inclination to perform microbiological confirmation |

## Appendix 4: Good Practice and Ethical Considerations

**Monitoring/Audit**

A systematic and independent audit of surveillance systems should be undertaken to ensure that surveillance and surveillance-related activities were conducted following the relevant surveillance protocol, standard operating procedure (SOP), ethical guidelines, and regulatory requirement(s) established by local public health. Existing surveillance review tools can be modified to guide the investigation (e.g., WHO’s ‘[Tools for a surveillance review: Vaccine Preventable Diseases Surveillance Standards](https://www.who.int/publications/m/item/vaccine-preventable-diseases-surveillance-standards-annex1)’). Surveillance as part of a clinical study should adhere to the ICH Guidelines for Good Clinical Practice^1^.

**Quality Control and Quality Assurance**

A quality management plan should be written before the start of surveillance to establish and ensure the quality of processes, data, and documentation associated with surveillance activities. It encompasses both quality control (QC) and quality assurance (QA) activities.

Surveillance systems should develop a SOP to ensure confidentiality for all cases, ensure that clinical specimens and bacterial isolates obtained are not compromised by human and processing errors, validate data integrity, and maintain multiple layers of security. A SOP will ideally detail:

- Data storage. Including participants’ unique surveillance ID numbers in each respective dataset enables linkage to other datasets, such as hospital admissions, facilitating the capture of complications and ensuring that all personal identifying information is removed from research/surveillance datasets.
- Data evaluation for protocol compliance and source document accuracy.
- Document review (e.g., specimen tracking logs, questionnaires), who is responsible, and frequency.
- Who the responsible person is for addressing QA issues (correcting procedures that do not comply with the surveillance protocol) and QC issues (correcting errors in data entry).
- Staff training activities and processes for documenting surveillance staff training.
- Maintenance and strict adherence to surveillance delegation log (list of staff involved in the surveillance and their duties/roles).
- Clinical and laboratory SOP and accreditation.
- Regular audits of surveillance data to ensure accuracy and completion.
- System for periodic and refresher training for surveillance team.

**Ethics of Surveillance**

The global network of WHO Collaborating Centres for Bioethics in collaboration with the U.S. Centers for Disease Control and Prevention developed ethical guidelines for public health surveillance, including common good, respect for persons, and good governance. The guidelines cover the (i) broad responsibility for undertaking surveillance and subjecting it to ethical scrutiny; (ii) obligation for ensuring appropriate protection and rights; (iii) considerations in making decisions about how to communicate and share surveillance data. The guidelines are available at <https://apps.who.int>. Countries should implement these guidelines and monitor them regularly. As appropriate, surveillance protocols should adhere to existing country-specific ethical guidelines.

^1^U.S. Department of Health and Human Services. *E6(R2) Good Clinical Practice: Integrated Addendum to ICH E6(R1) Guidance for Industry* Maryland 2018.

## Appendix 5: Definitions of Key Surveillance Terms

| **Syndromic surveillance** | Syndromic surveillance refers to the use of a clinical syndrome – a constellation of symptoms and signs – as the case definition for detection of suspect cases. Syndromic surveillance can be used for initial case detection, but laboratory confirmation should occur to increase the accuracy of the system^1^. |
| --- | --- |
| **Active surveillance** | Active case detection means that designated public health surveillance staff actively detect cases and report to the public health system ^1^. |
| **Passive surveillance** | Passive case detection means that health facility staff detect and report cases to the public health system^1^ |
| **Facility-based surveillance** | Facility-based surveillance is based on ascertainment of cases in persons who seek care at health facilities, including outpatient clinics, doctors’ offices, hospitals and emergency departments ^1^. |
| **Sentinel-site surveillance** | Sentinel-site surveillance refers to a system that captures cases at one or more specialized sites, such as hospitals, clinics, schools or pharmacies^1^. |
| **Community-based surveillance** | Community-based surveillance is the systematic detection and reporting of events of public health significance within a community-by-community members. Community-based surveillance enables earlier detection of the disease of interest and captures illnesses in persons who do not seek care in a hospital^2^ |
| **Population-based surveillance** | Population-based surveillance attempts to capture all cases in a well-defined catchment population (for example, the entire population of a country). |
| **Healthcare utilization surveys** | Healthcare utilization surveys characterize the health care-seeking behavior of ill persons by describing where ill persons sought health care for their illnesses, and soliciting reasons for not seeking health care ^3^ |
| **Unique identifier** | Unique identifiers are unique numbers or numbers and letter combinations that are allocated to a specific individual person. |

^1^World Health Organization. Surveillance standards for vaccine-preventable diseases. 2018.

^2^World Health Organization. A definition for community-based surveillance and a way forward: results of the who global technical meeting, france, 26 to 28 june 2018. *Eurosurveillance.* 2019;24(2).

^3^Deutscher M, Van Beneden C, Burton D, et al. Putting surveillance data into context: the role of health care utilization surveys in understanding population burden of pneumonia in developing countries. *Journal of epidemiology and global health.* 2012;2(2):73-81.

## Appendix 6: Echocardiographic Screening for Rheumatic Heart Disease (RHD)

While full echocardiographic assessment by a trained cardiologist for every individual in the survey population would produce the most accurate estimate of disease prevalence, this approach is typically not feasible logistically or financially or is limited by the available skilled workforce. Most screening studies have undertaken echocardiography with a portable, handheld device, followed by confirmatory echocardiography with a fully functional machine capable of continuous-wave Doppler (a necessary component to fulfill WHF criteria). Task shifting to non-expert healthcare workers is effective and should be considered by investigators implementing screening programs^1-6^.

A brief examination concentrating on the mitral and aortic valves can give sufficient information to diagnose and grade the severity of RHD lesions and takes about 10 minutes per person. Until the best strategy for training, including standardized competency assessments and accreditation processes, is determined, the decision regarding which model to adopt falls on investigators and healthcare systems implementing active surveillance. The following components should be considered when choosing the best training strategy: available human resources, previous ultrasound experience of those to be trained, number to be screened, and time allocated to screening. Regardless of which model is adopted, all who implement screening programs must ensure that those performing the screening echocardiography have demonstrated baseline and continuing competency.

Based on available data and practical considerations, we recommend a two-part echocardiographic screening strategy. Following a positive screening echocardiogram, a confirmatory echocardiogram should be performed, including parasternal long axis and apical four chamber views, noting valve morphology on cross-sectional two-dimensional imaging and the degree and extent of mitral and aortic regurgitation using color flow Doppler. Continuous-wave Doppler is used to measure the peak velocity of the affected valve(s) and transvalvular flow. Views need not be taken of the tricuspid and pulmonary valves unless there is severe disease of mitral or aortic valves. Such views may pick up other major pathology (e.g., congenital heart disease) but is of little utility when diagnosing RHD. The presence of pulmonary hypertension should be assessed in the setting of mitral stenosis, either through standard interrogation of tricuspid valve regurgitant velocity (if present), or through interrogation of pulmonary insufficiency velocity (if present). Lastly, the severity of valvular disease of the affected valve(s) should be assessed using the 2014 AHA/ACC Guideline for the Management of Patients with Valvular Heart Disease^1^.

If the primary aim of screening echocardiography is to provide data for an intervention (e.g., sore throat programs^2^ or vaccine) study, then before and after methodology should be identical. If new methods have been developed between the baseline and post-intervention period, the new and old methods should be used to ensure before and after comparability while incorporating the latest technology and standards.

^1^Engelman D, Kado JH, Reményi B, et al. Focused cardiac ultrasound screening for rheumatic heart disease by briefly trained health workers: a study of diagnostic accuracy. *Lancet Glob Health.* 2016;4(6):e386-394.

^2^DeWyer A, Scheel A, Otim IO, et al. Improving the accuracy of heart failure diagnosis in low-resource settings through task sharing and decentralization. *Glob Health Action.* 2019;12(1):1684070.

^3^ Mirabel M, Bacquelin R, Tafflet M, et al. Screening for rheumatic heart disease: evaluation of a focused cardiac ultrasound approach. *Circ Cardiovasc Imaging.* 2015;8(1).

^4^Ploutz M, Lu JC, Scheel J, et al. Handheld echocardiographic screening for rheumatic heart disease by non-experts. *Heart.* 2016;102(1):35-39.

^5^Beaton A, Nascimento BR, Diamantino AC, et al. Efficacy of a Standardized Computer-Based Training Curriculum to Teach Echocardiographic Identification of Rheumatic Heart Disease to Nonexpert Users. *The American Journal of Cardiology.* 2016;117(11):1783-1789.

^6^Francis JR, Whalley GA, Kaethner A, et al. Single-View Echocardiography by Nonexpert Practitioners to Detect Rheumatic Heart Disease: A Prospective Study of Diagnostic Accuracy. *Circulation: Cardiovascular Imaging.* 2021;14(8):e011790.

## Appendix 7: Administrative Health Databases

Administrative data from laboratory datasets and electronic medical records (EMRs) from primary healthcare and emergency departments covering whole communities can provide a timely and cost-effective surveillance option.

An important consideration when using EMRs to calculate disease estimates in a population is that the data are collected and coded as part of service delivery rather than for surveillance purposes. As such, EMRs are often prone to missing data on key fields and require the conversion of unstructured/narrative text, which can be resource-intensive and subjective. For EMRs that include or rely on free text, new methods in machine learning or deep learning could improve case identification^1,2^. Further, data are limited to patients who attend health services, and are subject to variance in physician’s propensity to seek microbiological confirmation, which may be subject to bias (e.g., more severe infections, more clinically ambiguous, one not responding to treatment) and underestimate disease incidence. However, an advantage to administrative data is that, in well-established systems, data are collected systematically, well-structured and are often population-based. EMRs can form the basis of enhanced surveillance by using an additional data collection form to augment routinely collected data.

Routinely collected clinic data may be insufficient for evaluating potential cases against the full criteria required to meet surveillance case definitions, especially when microbiological testing is not routinely conducted or recorded. Further, the data may be insufficient for addressing other surveillance objectives, such as variant typing and antimicrobial susceptibility testing.

^1^Ayala Solares JR, Diletta Raimondi FE, Zhu Y, et al. Deep learning for electronic health records: A comparative review of multiple deep neural architectures. *J Biomed Inform.* 2020;101:103337.

^2^Wang S, Lengeler C, Mtasiwa D, et al. Rapid Urban Malaria Appraisal (RUMA) II: epidemiology of urban malaria in Dar es Salaam (Tanzania). *Malar J.* 2006;5.

## Appendix 8: Variables for Inclusion in Rheumatic Heart Disease (RHD) Surveillance Datasets

| **Category of variables** | **Required variables** | **Optional variables** |
| --- | --- | --- |
| General | - Unique ID number - Date of enrolment | - Enrolment site |
| Demographics | - Age in years - Gender | - Date of birth - Race/ethnicity - Residential address |
| Medical history | History of:   - ARF: Definite, Probable or No - Date of RHD diagnosis | - Date of last ARF episode - Previous valve replacement surgery, including year and location - Complications of RHD and date of occurrence (stroke, endocarditis, atrial fibrillation |
| Classification and severity of disease | - Date of most recent echo - 2012 WHF Category   - - Definite (A,B, C,D) (age-dependent)     - Borderline (A,B,C) (for individuals <20 years)     - Mitral valve morphology       - AMVL thickening >3 mm (age-specific)       - Chordal thickening       - Restricted leaflet motion       - Excessive leaflet tip motion during systole     - Aortic valve morphology       - Irregular or focal thickening       - Coaptation defect       - Restricted leaflet motion       - Prolapse - Jet length of MR/AR | - NYHA class - *Severity of valve lesions   - - MR – Mild/Mod/Severe     - AR – Mild/Mod/Severe     - MS – Mild/Mod/Severe     - AS – Mild/Mod/Severe - If AS present:   - - Mean AS gradient     - Aortic valve area - If MS present:   - - Mean MS gradient     - Mitral valve area |
| Medications | - Secondary prophylaxis: - BPG 2/3/4 weekly - Oral Pen V - Erythromycin - Other (specify) | - Estimated adherence - Warfarin and INR target - Heart failure medications (specify) |
| Diagnostics | - Date of most recent echo | - EKG (rhythm, ventricular rate, additional findings) |

*Refer to Horton et al for further reading on classifying the severity of valvular lesions

Horton A, Gentles T, Remenyi B. Chapter 5 - Clinical Evaluation and Diagnosis of Rheumatic Heart Disease. In: Dougherty S, Carapetis J, Zühlke L, Wilson N, eds. *Acute Rheumatic Fever and Rheumatic Heart Disease.* San Diego (CA): Elsevier; 2021:69-106.
